# Walking Speed Assessed by 4-Meter Walk Test in the Community-Dwelling Oldest Old Population in Vietnam

**DOI:** 10.3390/ijerph19169788

**Published:** 2022-08-09

**Authors:** Anh Trung Nguyen, Huong Thi Thu Nguyen, Huong Thi Thanh Nguyen, Thanh Xuan Nguyen, Tam Ngoc Nguyen, Thu Thi Hoai Nguyen, Anh Lan Nguyen, Thang Pham, Huyen Thi Thanh Vu

**Affiliations:** 1 Department of Geriatrics, Hanoi Medical University, Hanoi 100000, Vietnam; 2 Scientific Research Department, National Geriatric Hospital, Hanoi 100000, Vietnam; 3 Dinh Tien Hoang Institute of Medicine, Hanoi 100000, Vietnam; 4 Physiology Department, Hanoi Medical University, Hanoi 100000, Vietnam

**Keywords:** gait speed, health outcomes, community, old population, Vietnam

## Abstract

This study aims to provide data on usual walking speed in individuals aged 80 years or older and determine the association between walking speed and related factors in community-dwelling older adults. A cross-sectional study design was conducted to measure walking speed on community-dwelling elders aged 80 years or older in Soc Son district, Vietnam. Walking speed was assessed by a 4-Meter Walk Test with a usual-pace walking mode. Health-related characteristics of participants including risk of falls (The Timed Up and Go test, activities of daily living (ADL), instrumental activities of daily living (IADLs), cognitive impairment (Mini-Cog test) and frailty syndrome (The Reported Edmonton Frail Scale (REFS)). Multiple logistic regression was used to analyze the association between a slow walking speed and selected factors. A total of 364 older people were recruited, and the majority were female (65.4%). The overall average walking speed was 0.83 ± 0.27 m/s. The proportion of participants with a slow walking speed (<0.8 m/s) was 40.4%. Multiple logistic regression analyses showed that age, female, high fall risk (assessed by TUG test), ADL/IADL dependence and frailty syndrome had a negative effect on slow walking speed in this population. The results could provide useful reference data for further investigations and measures in clinical practice.

## 1. Introduction

Assessment of functional limitations and physical performance has been used to predict health outcomes in the old population. Walking speed, or gait speed, is among the most widely used measurements of functional mobility and performance of activities of daily living, especially for older adults [1]. It has been described as a quick, easy to perform, inexpensive, valid and reliable assessment [2]. A slow walking speed has been shown to correlate with hospitalization, declines in physical health, cognitive impairment, increased level of dependency, falls and mortality in older adults [3,4]. Moreover, it has been used to indicate the health outcome in rehabilitation, the onset of disability or frailty [5]. Walking speed has been described as the “sixth vital sign” with the potential to serve as a core indicator of health and function in ageing and disease [6]. A decrease in walking speed may be an early sign of any problems in one or many body systems.

Walking speed tests can be determined by different protocols with walking speed distances ranging from 2 m to 500 m in community-dwelling people aged 60 years or over [4,7]. The 4, 6, and 10 Meter Walk Tests (MWT) are some of the most commonly used methods to evaluate walking speed over a short distance in the clinical geriatric settings [6,8]. In healthy older adults, both the 4M and the 10MWT were shown to have high test–retest reliability and validity in measuring walking speed [9]. In particular, the 4MWT requires a stopwatch and a facility hallway, is simple and can be performed by non-professional trained staff.

Walking speed value depends on age, gender, diagnoses, and coordination among the musculoskeletal, neuromuscular and cardiovascular systems. It also depends on cognition and mental health [6]. Previous studies indicated that average self-selected walking speed for healthy subjects aged 60 years or older ranges from 0.6 to 1.45 m/s [10,11]. In non-disabled, community-dwelling older adults, a slow walking speed (less than 0.8 m/s) has been shown to correlate with several health-related outcomes including increased fall risk, ischemic stroke, depressive symptoms and incontinence while the 1.0 m/s cut-point has been used to predict mortality [4]. However, data are lacking on the healthy, community-dwelling, very old population.

Vietnam has been facing an aging population, along with comorbidities, geriatric syndromes and those burdens. Data from GSO (2010) indicate that the old population (aged 60 and over) as a percentage of the total population was from 8.69% in 2009 to 26.1% in 2049 [12]. In addition, the oldest old population group (80 years or older) is rapidly increasing. The 4MWT, which took less than two minutes to perform, may be a feasible outcome tool for the old population and suitable for the primary health care system in Vietnam. However, to date, there have been no published data on walking speed in older people in Vietnam. Therefore, the purpose of this study was to provide data on usual walking speed in individuals aged 80 years or older and determine the association between walking speed and related factors in community-dwelling older adults.

## 2. Materials and Methods

### 2.1. Design and Participants

A cross-sectional study design was used to analyze the result of the 4MWT on community-dwelling people aged 80 years or older in Soc Son district, Hanoi, Vietnam in 2018. This is a suburban area located in northern Hanoi with a population of approximately 340,000 people. This is a non-commercial agriculture area with ease of access to medical services.

Community-dwelling subjects aged ≥80 years were included in this study. The sample size was calculated by a formula with a confidence level of 0.05, and the expected prevalence of a slow walking speed in the community was 56.4% [13]. The minimum sample size was 312. It is expected that the number of people who agree to participate is 50%. With the estimated number of people aged 80 years or older in Soc Son district being 6462, the systematic random sampling method was performed with a sample interval k = 10. According to the list of local older people aged ≥ 80 years old, starting from the person with the order of 02, we choose one person every 15 people to invite to participate in this study. A total of 646 invitation letters were sent. Participants who accepted will come to the local clinics where they were screened for exclusion criteria. Participants were not included in this study if they were unable to perform the specific functional test (bedridden people, blind, hearing loss) or unable to communicate (severe cognitive impairment) or those with motor impairment resulted from acute diseases such as recent stroke, coronary heart disease, and cardiomyopathy or with acute musculoskeletal diseases or orthopedic diseases that might interfere with the walking speed. A total of 383 people came (59.3%) and a total of 364 participants completed the questionnaire and assessment.

### 2.2. Data Collection

Data were collected using an interviewer structured questionnaire on general characteristics (age, gender, educational levels and living status) from the participants and performed the functional tests. Comorbidities (hypertension, osteoarthritis of the knee and lumbar spine) were collected through interview, clinical examination and doctor’s diagnosis. Medical staff who conducted data included five well-trained geriatric nurses and nursing students.

### 2.3. Measurements

#### 2.3.1. The 4-Meter Walk Test

Walking speed was assessed by the 4MWT with a usual-pace walking mode [14]. The main equipment for measuring was a stopwatch, tape to mark start and end points. To prepare for the test, participants were asked to wear comfortable clothes and footwear suitable for walking and not performing vigorous activities for 2 h before testing. A 4 m distance was measured and marked the starting and ending points on the floor. The nurse first demonstrated the test. Next, the participant performed a trial. Participants were instructed to walk at usual pace from a standing position that was normal and comfortable for them until they crossed the finish line. Time was recorded from the first foot movement and stop timing when the participant’s foot made contact with the floor at the end of the walking course. The 0.8 m/s cut-point was used to represent a slow walking speed [4]. Height-normalized walking speed was calculated by dividing walking speed with body height.

#### 2.3.2. Other Health-Related Measures

Weight and height were measured and used to calculate the body mass index (BMI) using the formula weight/height^2^ (kg/m^2^). BMI was categorized into three groups: underweight (<18.5), normal (18.5–22.9), overweight and obesity (≥23) [15].

Risk of falls: The Timed Up and Go test (TUG) was used to indicate the risk of falls among this population. Participants were asked to rise from a 45-cm chair, walk three meters with their usual walking pace, turn around, walk back to the chair and sit down. Timing started when the participants rose from their chairs and ended when they completely sat back down. The test was repeated twice and the final time was the average of the two trials. Those completing the test ≥12 s were classified into the “high fall risk” group [16].

Functional dependence: The Katz and Lawton scales were used to evaluate individuals’ functional ability in the performance of activities of daily living with or without instruments (IADL and ADL, respectively). A total score of <6 for the ADL scale and <8 for the IADL scale represents a dependence in daily functional activities or daily functional activities with an instrument, respectively [17,18].

Cognitive impairment was assessed using the Mini-Cog test. The test consists of a 3-word recall and clock drawing test. One point for each word recalled, for a 3-word recall score 1, 2 or 3. Two points for a normal clock or 0 (zero) points for an abnormal clock drawing. For this study, if the total score of 3-word recall and clock drawing test was <3, a participant was categorized as having cognitive impairment [19]. People with severe cognitive impairment were excluded from this study.

Frailty syndrome was assessed using the Reported Edmonton Frail Scale (REFS). The REFS involves nine frailty domains (cognition, general health status, functional independence, social support, medication use, nutrition, mood, continence and functional performance). With a maximum score of 18, frailty was divided into 2 levels: non-frail (0–7 scores) and frail (8–17 scores) [20].

### 2.4. Statistical Analysis

All data analysis used the SPSS software version 16.0. Characteristic variables were expressed in numbers and percentages as the mean (and standard deviations, SD). Walking speed measures were converted to meters per second (m/s). T-test and the Chi-squared test were used to measure relationships between walking speed, prevalence of a slow walking speed and other characteristics. A *p*-value < 0.05 was considered a statistical significance. Multivariate logistic regression was employed to identify the potential factors associated with a slow walking speed. Univariate logistic regression was performed on general factors (age, gender, BMI); stroke characteristics and other potential factors that can be associated with a slow walking speed based on the literature such as cognitive impairment, high fall risk, ADL and IADL dependence and frailty. Only variables that had a *p*-value < 0.20 on univariate analysis were selected for multivariate analysis.

### 2.5. Ethical Approval

The study protocol was approved by the institutional review board of the National Geriatric Hospital (Reference number: 1337/QD-BVLKTW). All participants were asked to give their oral informed consent and they could withdraw anytime. Their information was kept confidential and used only for research purposes.

## 3. Results

Among 364 participants aged 80 years old or older, the mean age was 84.5 (SD = 4.2) years old. The majority were female (65.4%), living with family (94.8%), low educational level (92.9%) and normal BMI (52.2%). The prevalence of hypertension and osteoarthritis of knee and lumbar spine was 29.9% and 48.4%, respectively. Table 1 depicts that the prevalence of high fall risk, ADL dependence, IADL dependence, mild to moderate cognitive impairment and frailty syndrome was 43.7%, 27.5%, 82.3%, 37.6% and 10.4%, respectively.

Walking speed and height-normalized walking speed values are shown in Table 2. The overall mean for usual walking speed in this population was 0.83 ± 0.27 m/s (95% CI: 0.8–0.86). The mean walking speed in males (0.93 ± 0.26 m/s) was higher than that in females (0.78 ± 0.26 m/s) with *p* < 0.001. Walking speed decreased with age. Walking speed in the group with high fall risk, ADL and IADL dependence and frailty was statistically significantly lower than the group without these conditions (*p* < 0.001).

Distribution of comorbidities (hypertension and osteoarthritis) between group of gender, age and walking speed was showed in Table S1 (Supplementary Material). There were no statistically significant differences between walking speed and height-normalized walking speed in these subgroups.

Table 3 shows the proportion of very old people with a slow walking speed (<0.8 m/s). Overall, the proportion of participants with a slow walking speed was 40.4%, of which the percentage was higher in female (48.3%) than in male (25.4%) with *p* < 0.001. The rate of a slow walking speed increased with age and was higher in the group with cognitive impairment, high fall risk, ADL and IADL dependence and frailty with *p* < 0.05.

Table 4 shows the results from multiple logistic regression analyses of the relationship between a slow walking speed and some factors. Age ≥ 90 years old, female, high fall risk (assessed by TUG test), ADL/IADL dependence and frailty syndrome had a negative effect on a slow walking speed in this population.

## 4. Discussion

The main outcomes of this study are to provide data on usual walking speed in individuals aged 80 years or older and determine the association between walking speed and demographic and health-related factors in community-dwelling older adults. The mean usual walking speed found in this study was 0.83 ± 0.27 (m/s). A slow walking speed (<0.8 m/s) was associated with advanced age, female gender, high fall risk, functional dependence and frailty.

In our investigation, evaluation of the very old population may lead to a slower walking speed compared to previous literature [11,21,22,23]. The results of our calculations showed that the average normative walking speed in all subjects is 0.83 m/s (95% CI = 0.8–0.86); 0.93 m/s for males and 0.78 m/s for females. Compared with normal walking speed in the meta-analysis for males and females aged 80–89 years, which was approximately 0.97 m/s for males and 0.94 m/s for females, our study showed a slower walking speed in both males and females [11]. This could be due to the different study populations between a Southeast Asia country and European and American countries. Indeed, our results were similar to those reported in 80–99 year-old Japanese (0.92 m/s in males and 0.80 m/s in females) [22]. Our finding could be explained by the different study populations in terms of higher average age and complicated health status. Older people are facing immobility and functional decline not only because of the burden from chronic diseases due to the natural occurrence of advanced age but also from social-economic and lifestyle changes. The reference values for older people aged 80 and over in Vietnam help to identify older people with a slow walking speed, an additional reference for the old and oldest groups, and to establish specific strategies to lower the risk of adverse outcomes.

In this study, walking speed and height-normalized speed decreased with age and was higher in males than in females, which is in accordance with previous research [11,23,24,25,26]. Declined lower limb muscle function and fewer muscle fibers in female compared with male along with aging process and the attribution of the larger step and stride length in male may be the cause of this condition [27,28]. Other potential determinants have been proposed to account for gender differences in walking speed such as a greater change toward smaller fibers in females during aging-related atrophy [29], a substantial decrease in estrogen, a potent hormone improving the intrinsic quality of skeletal muscle [30], body composition [31] or lower level of physical activity [32]. Our study showed that a slow walking speed was significantly associated with high fall risk, ADL/IADL dependence and frailty syndrome. This result was similar to previous studies [33,34]. Reduce walking speed is altered with ageing, but impaired balance and stability, lower extremity strength are also contributors to gait change [35]. This can lead to difficulties in daily activities, functional dependence and increased level of dependence and risk of fall in very old population [34,36,37,38,39,40]. In addition, impaired gait speed in frail older adults has been documented in the literature [41,42,43]. For instance, in a cross-sectional study examining the association between gait variability and frailty, it was found that frailty was associated with higher variability for all the gait parameters [44]. Thus, our finding suggests that walking speed should be improved by the lifestyle to strengthen muscles of the lower extremities in daily life. Further, early detection of a slow walking speed by using appropriate, reliable and accurate techniques during the comprehensive geriatric assessment, in order to indicate future demand for supporting and caring in the Vietnamese oldest population.

The implications of this article indicate that normal walking speed should be further integrated with physical screenings, assessments, and used as an outcome measure. The assessment of gait speed over a short distance, such as the 4 m walk, should be the choice because it has been demonstrated to be feasible in the community as well as in clinical settings. In clinical settings where numerous patients are measuring the walking speed, the 4MWT was found positive response both in time to train and the overall burden for clinical assistants in administering the test. Several cut-points for gait speed as predictors of adverse outcomes have been suggested depending on the length of track, outcome, settings and assessed population. A 4 m walking speed lower than 0.8 m/s is commonly used to predict health adverse outcomes [4,45,46,47,48]. This finding provided cut-point gait speed on the oldest old community-dwelling population and it might be a useful threshold to identify the risk of further functional decline in clinical practice, using a 4MWT.

To our knowledge, for the first time, the walking speed values for 80 years old and older of a community population in the North of Vietnam are presented, providing reference values for screening tests. However, there are some limitations to this study. Firstly, the cross-sectional nature of this study does not allow conclusions about the predictability of health outcomes of walking speed. Secondly, it was carried out in one geographic region in Vietnam despite the diverse sample information. A standardized procedure is needed in future research, gold standard measurements of gait speed assessment, such as instrumented walkways and stereo-photogrammetry were not used in the present study. Gait aids were not included in the models because of the lack of detail in reported walk test protocols. Still, the result obtained can be used as a reference for other community-based studies in Vietnam and provides evidence for screening gait speed in very old population.

## 5. Conclusions

This study revealed the reference data of usual walking speed in 80 years and over community-dwelling older adults in rural areas in Vietnam. Our result indicated the low values of normal walking speed observed in this population compared to other European and American countries. Walking speed decreased with advanced age, female gender, high fall risk, ADL/IADL dependence and frailty syndrome. Further validations of the test in institutionalized and hospitalized older people and long-term studies on walking speed and adverse health outcomes are needed.

## Figures and Tables

**Table 1:** Characteristics of the study participants (n = 364).

	Frequencies	Percentages
**Age groups**		
80–84 years	209	57.4
85–89 years ≥90 years	109 46	30.0 12.6
**Gender**		
Male	126	34.6
Female	238	65.4
**Educational level**		
High	26	7.1
Low	338	92.9
**Living alone**		
No	345	94.8
Yes	19	5.2
**Body mass index**		
<18.5 kg/m^2^	115	31.8
18.5–22.9 kg/m^2^	189	52.2
≥23 kg/m^2^	58	16.0
**Comorbidities**		
Hypertension	109	29.9
Osteoarthritis	176	48.4
**High fall risk**		
No	205	56.3
Yes	159	43.7
**ADL dependence**		
No	264	72.5
Yes	100	27.5
**IADL dependence**		
No	68	18.7
Yes	296	81.3
**Mild to moderate cognitive impairment**		
No	227	62.4
Yes	137	37.6
**Frailty syndrome**		
No	326	89.6
Yes	38	10.4
	**Mean (SD)**	**Min–Max**
**Age (years)**	84.5 (4.2)	80–105
**Height (cm)**	148.2 (8.0)	126.0–172.3
**Weight (kg)**	44.0 (8.1)	25.7–80.0
**BMI (kg/m^2^)**	20.0 (3.1)	12.2–40.8

**Table 2:** Walking speed and height-normalized walking speed values among this study population (n = 364).

	Walking Speed (m/s)		Height-Normalized Speed (m/s)	
	Mean (SD)	95% CI	Mean (SD)	95% CI
**Overall**	0.83 (0.27)	0.80–0.86	0.56 (0.17)	0.54–0.58
**Age groups**				
80–84 years	0.89 (0.26)	0.85–0.92	0.59 (0.17)	0.57–0.61
85–89 years	0.81 (0.26)	0.76–0.85	0.54 (0.17)	0.51–0.57
≥90 years	0.66 (0.26) ^a^	0.58–0.74	0.45 (0.18) ^a^	0.40–0.50
**Gender**				
Male	0.93 (0.26)	0.88–0.97	0.60 (0.16)	0.57–0.63
Female	0.78 (0.26) ^a^	0.75–0.81	0.54 (0.18) ^a^	0.52–0.56
**Body mass index**				
<18.5 kg/m^2^	0.81 (0.27)	0.76–0.86	0.54 (0.17)	0.51–0.57
18.5–22.9 kg/m^2^	0.85 (0.27)	0.81–0.89	0.57 (0.17)	0.55–0.59
≥23 kg/m^2^	0.82 (0.26)	0.75–0.89	0.55 (0.21)	0.46–0.64
**Hypertension**				
No	0.84 (0.27)	0.80–0.87	0.56 (0.17)	0.54–0.58
Yes	0.82 (0.27)	0.77–0.87	0.55 (0.17)	0.52–0.59
**Osteoarthritis**				
No	0.83 (0.28)	0.78–0.87	0.55 (0.18)	0.53–0.58
Yes	0.84 (0.25)	0.80–0.86	0.56 (0.16)	0.54–0.59
**High fall risk**				
No	0.96 (0.22)	0.93–0.98	0.63 (0.14)	0.61–0.65
Yes	0.68 (0.25) ^a^	0.64–0.71	0.48 (0.17) ^a^	0.45–0.50
**ADL dependence**				
No	0.87 (0.25)	0.84–0.90	0.64 (0.14)	0.61–0.67
Yes	0.73 (0.29) ^a^	0.67–0.78	0.59 (0.16) ^a^	0.56–0.61
**IADL dependence**				
No	0.93 (0.22)	0.88–0.98	0.64 (0.14)	0.61–0.67
Yes	0.81 (0.28) ^a^	0.78–0.84	0.53 (0.18) ^a^	0.51–0.56
**Mild to moderate cognitive impairment**				
No	0.86 (0.25)	0.82–0.89	0.58 (0.17)	0.56–0.60
Yes	0.79 (0.29)	0.74–0.84	0.53 (0.18) ^a^	0.50–0.56
**Frailty syndrome**				
No	0.86 (0.26)	0.83–0.89	0.58 (0.17)	0.56–0.59
Yes	0.59 (0.22) ^a^	0.52–0.66	0.41 (0.15) ^a^	0.36–0.46

**Notes:**^a^*p* < 0.05.

**Table 3:** Prevalence of a slow walking speed among the study participants (n = 364).

	Slow Walking Speed (Walking Speed < 0.8 m/s)		
	Frequencies	Percentages	95% CI
**Overall**	147	40.4	35.3–45.6
**Age groups**			
80–84 years	67	32.1	25.8–38.8
85–89 years	49	45.0	35.4–54.8
≥90 years	31	67.4 ^a^	52.0–80.5
**Gender**			
Male	32	25.4	18.1–33.9
Female	115	48.3 ^a^	41.8–54.9
**Body mass index**			
<18.5 kg/m^2^	54	47.0	37.6–56.5
18.5–22.9 kg/m^2^	68	36.0	29.1–43.3
≥23 kg/m^2^	24	41.4	28.6–55.1
**Hypertension**			
No	106	41.6	35.5–47.6
Yes	41	37.6	28.4–47.0
**Osteoarthritis**			
No	75	39.9	32.8–46.6
Yes	72	40.9	33.2–48.3
**High fall risk**			
No	42	20.5	15.2–26.7
Yes	105	66.0 ^a^	58.1–73.4
**ADL dependence**			
No	87	33.0	27.3–39.0
Yes	60	60.0 ^a^	49.7–69.7
**IADL dependence**			
No	16	23.5	14.1–35.4
Yes	131	44.3 ^b^	38.5–50.1
**Mild to moderate cognitive impairment**			
No	81	35.7	29.5–42.3
Yes	66	48.2 ^b^	39.6–56.9
**Frailty syndrome**			
No	117	35.9	30.7–41.4
Yes	30	79.0 ^a^	62.7–90.4

**Notes:**^a^ *p* < 0.001, ^b^ *p* < 0.05. Abbreviations: ADL, activities of daily living; IADL, instrumental activities of daily living.

**Table 4:** Multiple logistic regression analyses of the association between a slow walking speed and selected factors.

Dependent Variables: Slow Walking Speed (Walking Speed < 0.8 m/s)		
Independent Variables	Odds Ratio	95% CI
**Age groups**		
80–84 years	1	-
85–89 years	1.6	0.9–2.9
≥90 years	3.3	1.5–7.3
**Gender**		
Male	1	-
Female	2.5	1.4–4.3
**Body mass index**		
<18.5 kg/m^2^	1	-
18.5–22.9 kg/m^2^	0.6	0.4–1.1
≥23 kg/m^2^	0.9	0.4–1.8
**High fall risk**		
No	1	-
Yes	5.8	3.5–9.6
**ADL/IADL dependence**		
No	1	-
Yes	3.0	1.2–7.4
**Mild to moderate cognitive impairment**		
No	1	-
Yes	1.1	0.6–1.8
**Frailty syndrome**		
No	1	-
Yes	2.8	1.1–7.3

**Notes:** ADL/IADL dependence: patients had a dependency on activities of daily living (ADL) or instrumental activities of daily living (IADL).

## Data Availability

The datasets of this study are available from the corresponding author on reasonable request.

## Supplementary Materials

The following supporting information can be downloaded at: https://www.mdpi.com/article/10.3390/ijerph19169788/s1, Table S1: Distribution of comorbidities (hypertension and osteoarthritis) between group of gender, age and walking speed.

## References

[1] Lusardi M., Pellecchia G., Schulman M. (2003). Functional performance in community living older adults. J. Geriatr Phys. Ther..

[2] Cesari M., Kritchevsky S.B., Penninx B.W.H.J., Nicklas B.J., Simonsick E.M., Newman A.B., Tylavsky F.A., Brach J.S., Satterfield S., Bauer D.C. (2005). Prognostic value of usual gait speed in well-functioning older people--results from the Health, Aging and Body Composition Study. J. Am. Geriatr. Soc..

[3] Graham J.E., Ostir G.V., Fisher S.R., Ottenbacher K.J. (2008). Assessing walking speed in clinical research: A systematic review. J. Eval. Clin. Pract..

[4] Abellan van Kan G., Rolland Y., Andrieu S., Bauer J., Beauchet O., Bonnefoy M., Cesari M., Donini L.M., Gillette Guyonnet S., Inzitari M. (2009). Gait speed at usual pace as a predictor of adverse outcomes in community-dwelling older people an International Academy on Nutrition and Aging (IANA) Task Force. J. Nutr. Health Aging.

[5] Fairhall N., Aggar C., Kurrle S.E., Sherrington C., Lord S., Lockwood K., Monaghan N., Cameron I.D. (2008). Frailty Intervention Trial (FIT). BMC Geriatr..

[6] Fritz S., Lusardi M. (2009). White paper: “walking speed: The sixth vital sign”. J. Geriatr. Phys. Ther..

[7] Rydwik E., Bergland A., Forsén L., Frändin K. (2012). Investigation into the reliability and validity of the measurement of elderly people’s clinical walking speed: A systematic review. Physiother. Theory Pract..

[8] Studenski S., Perera S., Wallace D., Chandler J.M., Duncan P.W., Rooney E., Fox M., Guralnik J.M. (2003). Physical performance measures in the clinical setting. J. Am. Geriatr. Soc..

[9] Peters D.M., Fritz S.L., Krotish D.E. (2013). Assessing the reliability and validity of a shorter walk test compared with the 10-Meter Walk Test for measurements of gait speed in healthy, older adults. J. Geriatr. Phys. Ther..

[10] Oberg T., Karsznia A., Oberg K. (1993). Basic gait parameters: Reference data for normal subjects, 10–79 years of age. J. Rehabil. Res. Dev..

[11] Bohannon R.W. (1997). Comfortable and maximum walking speed of adults aged 20–79 years: Reference values and determinants. Age Ageing.

[12] The 2009 Vietnam Population and Housing Census: Major Findings. https://www.gso.gov.vn/en/data-and-statistics/2019/03/the-2009-vietnam-population-and-housing-census-major-findings/.

[13] Castell M.-V., Sánchez M., Julián R., Queipo R., Martín S., Otero Á. (2013). Frailty prevalence and slow walking speed in persons age 65 and older: Implications for primary care. BMC Fam. Pract..

[14] Maggio M., Ceda G.P., Ticinesi A., De Vita F., Gelmini G., Costantino C., Meschi T., Kressig R.W., Cesari M., Fabi M. (2016). Instrumental and Non-Instrumental Evaluation of 4-Meter Walking Speed in Older Individuals. PLoS ONE.

[15] WHO Expert Consultation (2004). Appropriate body-mass index for Asian populations and its implications for policy and intervention strategies. Lancet.

[16] Barry E., Galvin R., Keogh C., Horgan F., Fahey T. (2014). Is the Timed Up and Go test a useful predictor of risk of falls in community dwelling older adults: A systematic review and meta-analysis. BMC Geriatr..

[17] Shelkey M., Wallace M. (2000). Katz Index of Independence in Activities of Daily Living (ADL). Director.

[18] Lawton M.P., Brody E.M. (1969). Assessment of older people: Self-maintaining and instrumental activities of daily living. Gerontologist.

[19] Borson S., Scanlan J.M., Chen P., Ganguli M. (2003). The Mini-Cog as a screen for dementia: Validation in a population-based sample. J. Am. Geriatr Soc..

[20] Vu H.T.T., Nguyen T.X., Nguyen T.N., Nguyen A.T., Cumming R., Hilmer S., Pham T. (2017). Prevalence of frailty and its associated factors in older hospitalised patients in Vietnam. BMC Geriatr..

[21] Aoyagi K., Ross P.D., Nevitt M.C., Davis J.W., Wasnich R.D., Hayashi T., Takemoto T. (2001). Comparison of performance-based measures among native Japanese, Japanese-Americans in Hawaii and Caucasian women in the United States, ages 65 years and over: A cross-sectional study. BMC Geriatr..

[22] Nagasaki H., Itoh H., Hashizume K., Furuna T., Maruyama H., Kinugasa T. (1996). Walking patterns and finger rhythm of older adults. Percept. Mot. Skills.

[23] Bohannon R.W., Wang Y.-C. (2019). Four-Meter Gait Speed: Normative Values and Reliability Determined for Adults Participating in the NIH Toolbox Study. Arch. Phys. Med. Rehabil..

[24] Seino S., Shinkai S., Fujiwara Y., Obuchi S., Yoshida H., Hirano H., Kim H.K., Ishizaki T., Takahashi R., TMIG-LISA Research Group (2014). Reference values and age and sex differences in physical performance measures for community—Dwelling older Japanese: A pooled analysis of six cohort studies. PLoS ONE.

[25] McKay M.J., Baldwin J.N., Ferreira P., Simic M., Vanicek N., Wojciechowski E., Mudge A., Burns J., 1000 Norms Project Consortium (2017). Spatiotemporal and plantar pressure patterns of 1000 healthy individuals aged 3–101 years. Gait Posture.

[26] Kasović M., Štefan L., Štefan A. (2021). Normative Data for Gait Speed and Height Norm Speed in ≥ 60-Year-Old Men and Women. Clin. Interv. Aging.

[27] Lauretani F., Russo C.R., Bandinelli S., Bartali B., Cavazzini C., Di Iorio A., Corsi A.M., Rantanen T., Guralnik J.M., Ferrucci L. (2003). Age-associated changes in skeletal muscles and their effect on mobility: An operational diagnosis of sarcopenia. J. Appl. Physiol..

[28] Miller A.E., MacDougall J.D., Tarnopolsky M.A., Sale D.G. (1993). Gender differences in strength and muscle fiber characteristics. Eur. J. Appl. Physiol. Occup. Physiol..

[29] Callahan D.M., Miller M.S., Sweeny A.P., Tourville T.W., Slauterbeck J.R., Savage P.D., Maugan D.W., Ades P.A., Beynnon B.D., Toth M.J. (2014). Muscle disuse alters skeletal muscle contractile function at the molecular and cellular levels in older adult humans in a sex-specific manner. J. Physiol..

[30] Lowe D.A., Baltgalvis K.A., Greising S.M. (2010). Mechanisms behind estrogen’s beneficial effect on muscle strength in females. Exerc. Sport Sci. Rev..

[31] Tseng L.A., Delmonico M.J., Visser M., Boudreau R.M., Goodpaster B.H., Schwartz A.V., Simonsick E.M., Satterfield S., Harris T., Newman A.B. (2014). Body composition explains sex differential in physical performance among older adults. J. Gerontol. A Biol. Sci. Med. Sci..

[32] Sialino L.D., Schaap L.A., van Oostrom S.H., Picavet H.S.J., Twisk J.W.R., Verschuren W.M.M., Visser M., Wijnhoven H.A.H. (2021). The sex difference in gait speed among older adults: How do sociodemographic, lifestyle, social and health determinants contribute?. BMC Geriatr..

[33] Woo J., Ho S.C., Yu A.L. (1999). Walking speed and stride length predicts 36 months dependency, mortality, and institutionalization in Chinese aged 70 and older. J. Am. Geriatr. Soc..

[34] Shinkai S., Watanabe S., Kumagai S., Fujiwara Y., Amano H., Yoshida H., Ishizaki T., Yukawa H., Suzuki T., Shibata H. (2000). Walking speed as a good predictor for the onset of functional dependence in a Japanese rural community population. Age Ageing.

[35] Cruz-Jimenez M. (2017). Normal Changes in Gait and Mobility Problems in the Elderly. Phys. Med. Rehabil. Clin. N. Am..

[36] Wolfson L., Whipple R., Amerman P., Tobin J.N. (1990). Gait assessment in the elderly: A gait abnormality rating scale and its relation to falls. J. Gerontol..

[37] Montero-Odasso M., Schapira M., Soriano E.R., Varela M., Kaplan R., Camera L.A., Mayorga L.M. (2005). Gait velocity as a single predictor of adverse events in healthy seniors aged 75 years and older. J. Gerontol. A Biol. Sci. Med. Sci..

[38] Chu L.W., Chi I., Chiu A.Y.Y. (2005). Incidence and predictors of falls in the chinese elderly. Ann. Acad. Med. Singap..

[39] Navarro-Flores E., de Bengoa Vallejo R.B., Losa-Iglesias M.E., Palomo-López P., Calvo-Lobo C., López-López D., Martínez-Jiménez E.M., Romero-Morales C. (2020). The reliability, validity, and sensitivity of the Edmonton Frail Scale (EFS) in older adults with foot disorders. Aging (Albany NY).

[40] Navarro-Flores E., Romero-Morales C., Becerro de Bengoa-Vallejo R., Rodríguez-Sanz D., Palomo-López P., López-López D., Losa-Iglesias M.E., Calvo-Lobo C. (2020). Sex Differences in Frail Older Adults with Foot Pain in a Spanish Population: An Observational Study. Int. J. Environ. Res. Public Health.

[41] Lipsitz L.A. (2002). Dynamics of stability: The physiologic basis of functional health and frailty. J. Gerontol. A Biol. Sci. Med. Sci..

[42] Lipsitz L.A. (2004). Physiological complexity, aging, and the path to frailty. Sci. Aging Knowl. Environ..

[43] Binotto M.A., Lenardt M.H., Rodríguez-Martínez M.D.C. (2018). Physical frailty and gait speed in community elderly: A systematic review. Rev. Esc. Enferm. USP.

[44] Montero-Odasso M., Muir S.W., Hall M., Doherty T.J., Kloseck M., Beauchet O., Speechley M. (2011). Gait variability is associated with frailty in community-dwelling older adults. J. Gerontol. A Biol. Sci. Med. Sci..

[45] Cummings S.R., Studenski S., Ferrucci L. (2014). A diagnosis of dismobility—Giving mobility clinical visibility: A Mobility Working Group recommendation. JAMA.

[46] Inzitari M., Newman A.B., Yaffe K., Boudreau R., de Rekeneire N., Shorr R., Harris T.B., Rosano C. (2007). Gait speed predicts decline in attention and psychomotor speed in older adults: The health aging and body composition study. Neuroepidemiology.

[47] Cesari M., Onder G., Zamboni V., Manini T., Shorr R.I., Russo A., Bernabei R., Pahor M., Landi F. (2008). Physical function and self-rated health status as predictors of mortality: Results from longitudinal analysis in the ilSIRENTE study. BMC Geriatr..

[48] Rolland Y., Lauwers-Cances V., Cesari M., Vellas B., Pahor M., Grandjean H. (2006). Physical performance measures as predictors of mortality in a cohort of community-dwelling older French women. Eur. J. Epidemiol..

