# Supplementary material for: Walking Speed Assessed by 4-Meter Walk Test in the Community-Dwelling Oldest Old Population in Vietnam
Source: Int J Environ Res Public Health. 2022 Aug 9;19(16):9788. doi: 10.3390/ijerph19169788 (PMC9407834; doi:10.3390/ijerph19169788)
Supplement: Supplementary file 1 [file ijerph-19-09788-s001.zip › ijerph-1755412-supplementary.pdf]

International Journal of
Environmental Research and Public Health

MDPI

Table S1. Distribution of comorbidities (hypertension and osteoathritis) between group of gender, age and walking speed

Measure	Gender	Age	Hypertension		Osteoathritis	
			No	Yes	No	Yes
Walking speed (m/s)	Male	80 - 84	1.02 (0.24)	1.02 (0.21)	1.03 (0.23)	1.01 (0.23)
		85 - 89	0.87 (0.25)	0.91 (0.16)	0.91 (0.23)	0.86 (0.23)
		⩾ 90	0.73 (0.30)	0.64 (0.03)	0.68 (0.29)	0.79 (0.28)
Height-normalized walking speed (m/s)		80 - 84	0.65 (0.15)	0.65 (0.13)	0.66 (0.14)	0.64 (0.15)
		85 - 89	0.57 (9.16)	0.59 (0.10)	0.59 (0.15)	0.55 (0.14)
		≥ 90	0.48 (0.19)	0.43 (0.01)	0.46 (0.18)	0.52 (0.20)
Walking speed (m/s)	Female	80 - 84	0.84 (0.24)	0.80 (0.27)	0.83 (0.26)	0.83 (0.24)
		85 - 89	0.74 (0.28)	0.75 (0.26)	0.71 (0.27)	0.78 (0.27)
		⩾ 90	0.65 (0.24)	0.55 (0.29)	0.53 (0.28)	0.69 (0.22)
Height-normalized walking speed (m/s)		80 - 84	0.58 (0.16)	0.55 (0.18)	0.57 (0.18)	0.57 (0.16)
		85 - 89	0.51 (0.18)	0.52 (0.17)	0.50 (0.18)	0.53 (0.17)
		⩾ 90	0.46 (0.16)	0.39 (0.21)	0.37 (0.19)	0.49 (0.15)
